# Does Neighborhood Disadvantage Moderate the Relationship between Apolipoprotein E Status and Cognitive Functioning?

**DOI:** 10.1002/alz70857_103936

**Published:** 2025-12-25

**Authors:** Lyla Wadia, Stella Garriga, Timothy Belliveau, Franchesca Arias, Carmen Carrion

**Affiliations:** ^1^ Yale School of Medicine, New Haven, CT, USA; ^2^ University of Florida, Gainesville, FL, USA; ^3^ Yale University, New Haven, CT, USA

## Abstract

**Background:**

Research is inconclusive about the relationship between Apolipoprotein E (APOE) status and cognition, especially among APOE4 (ε4) heterozygotes. Environmental factors like neighborhood disadvantage are also associated with cognitive decline, however, few studies have integrated this with APOE research. This study seeks to address the gaps in literature by examining whether neighborhood disadvantage, defined by the Area Deprivation Index (ADI), moderates the relationship between APOE4 status and cognition.

**Method:**

This is a cross‐sectional analysis of data from the Connecticut Alzheimer's Disease Research Center (*N* = 190). Participants diagnosed with dementia or with two ε4 alleles were excluded. Adults over 50 years old (53.7% Female; *M*_education_=16.6 ± 2.56; 80.5% Non‐Hispanic White) (*M*_age_=72.6 ± 7.19) were cognitively healthy (62.6%) or diagnosed with MCI (37.4%). Participants completed a neuropsychological workup as part of their annual evaluation. Neuropsychological measures were grouped theoretically into attention, language, speed and executive function, memory, and visuospatial domains. Confirmatory factory analysis (CFA) was utilized to assure suitability to domains. Multiple linear regressions were used to evaluate main effects and interactions.

**Result:**

CFA results supported the domain groupings (Χ^2^(34)=95.395, *p* < .001; *RMSEA* = 0.080 [90% CI=0.062, 0.100]; *CFI* = 0.952; *TLI* = 0.922). Each linear regression included the following predictors and covariates: APOE status, ADI, ADI*APOE interaction, sex, race, education, age, and hypercholesterolemia. Only memory and attention domains met normality assumptions and neither the overall model for memory (*R^2^* = 0.17, *F*(10, 75)=1.48, *p* = .163) or attention (*R^2^* = 0.16, *F*(10, 75)=1.40, *p* = .199) were statically significant. APOE status was not a significant predictor for performance on memory (*β* = ‐0.36, *p* = .430) or attention (*β* = 0.52, *p* = .265) measures. ADI was not a significant predictor for performance on memory (*β* = 0.15, *p* = .593) or attention (β=0.24, *p* = .414) measures. The APOE*ADI interaction was not significant for measures of memory (*β* = ‐0.12, *p* = .486) or attention (*β* = ‐0.14, *p* = .429).

**Conclusion:**

In our subsample of participants, ADI did not moderate the relationship between APOE status and performance on attention or memory measures. However, research demonstrates a relationship between environmental factors and cognitive decline. Some factors remain modifiable, on an individual and policy level, and in order to shift towards a preventative rather than reactive care model, it is imperative to continue to explore modifiable risk factors (e.g., social determinants of health) that impact cognition.

